# Insights from the genetic and transcriptional characterization of a cancer of unknown primary (CUP)

**DOI:** 10.15252/emmm.202012685

**Published:** 2020-06-17

**Authors:** Veronica Davalos, Manel Esteller

**Affiliations:** ^1^ Josep Carreras Leukaemia Research Institute (IJC) Barcelona Catalonia Spain; ^2^ Centro de Investigacion Biomedica en Red Cancer (CIBERONC) Madrid Spain; ^3^ Institucio Catalana de Recerca i Estudis Avançats (ICREA) Barcelona Catalonia Spain; ^4^ Physiological Sciences Department School of Medicine and Health Sciences University of Barcelona (UB) Barcelona Catalonia Spain

**Keywords:** Cancer, Genetics, Gene Therapy & Genetic Disease

## Abstract

Cancer of unknown primary (CUP) defines a heterogeneous group of metastatic tumors that lack an identifiable primary tumor, despite a standardized diagnostic work‐up (Fizazi *et al*, 2015). CUPs are characterized by an aggressive clinical course, unusual metastatic pattern, and poor prognosis. Research in this field has been encouraged to unravel the complexity of this enigmatic entity and improve clinical management and survival of CUP patients. In this issue of *EMBO Molecular Medicine*, Benvenuti *et al* (2020) describe the molecular characterization of multiple synchronous and spatially distinct metastases from a CUP patient, shedding light on the evolutionary dynamic and distinctive features of CUP.

## Graphical abstract

M. Esteller and V. Davalos discuss the molecular characterization of multiple synchronous and spatially distinct metastases from a Cancer of Primary Unknown patient by P. Comoglio and colleagues, in this issue of *EMBO Mol Med*.

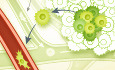

Despite the advances in medical imaging and molecular approaches, the primary site cannot be identified at the time of diagnosis in 1–2% of cancers (Rassy & Pavlidis, 2020). The existence of CUP as a real entity is further supported by cases where the primary tumor cannot be identified even after extensive diagnostic work‐up or exhaustive research of post‐mortem autopsy.

Clinical management of CUP patients is a challenge in the oncology field. Efforts to expand the therapeutic alternatives beyond empirical chemotherapy to improve survival have been centered on two different strategies: traditional tissue‐gnostic or tissue‐agnostic approaches. For the first, tumor‐type classification is the key to treatment allocation. Tissue specificity of gene expression and epigenetic profiles (Moran *et al*, 2016) has guided the development of tumor‐type classifiers for CUPs (Moran *et al*, 2017). Case reports, case series, and non‐randomized prospective studies of CUPs have indicated that tumor‐type specific therapies lead to improved outcomes; however, randomized controlled trials have not clearly demonstrated their superiority in comparison with empirical chemotherapy (Fizazi *et al*, 2019; Hayashi *et al*, 2019).

Considering that the tissue could no longer be the focus in CUPs, tissue‐agnostic approaches have also gained force in the last years. Molecularly driven therapies have already transformed the treatment landscape of several tumors harboring actionable mutations, independently of the tissue of origin. Along these lines, the ongoing phase II, randomized clinical trial CUPISCO (NCT03498521) will compare the efficacy of molecularly guided therapy versus standard platinum‐based chemotherapy in CUP cases.

Although the lack of a primary tumor in CUPs could be explained by limited sensitivity of imaging technologies for detecting very small tumors, or the possibility that the primary tumor regresses or remains dormant, the existence of metastases that develop following a distinctive biological pathway different to the canonical metastatic cascade remains to be explored (Fig 1).

**Figure 1:**
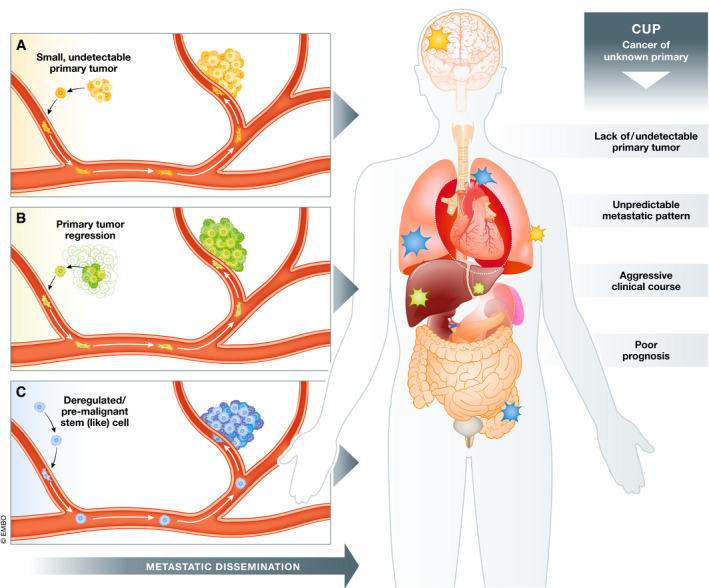
Hypothesized origins of CUP (A) Small primary tumors undetectable with available imaging technologies; (B) primary tumors that regress or remain dormant; (C) metastases that develop following a distinctive biological pathway, different to the canonical metastatic cascade of dissemination from a primary tumor, perhaps from cells with stem cell attributes.

In an effort to improve our knowledge on this enigmatic entity, Benvenuti *et al* (2020) describe in this issue of *EMBO Molecular Medicine* the molecular characterization of multiple synchronous and spatially distinct metastases from a CUP patient. A total of 15 metastases were retrieved at warm autopsy from nine different tissues of a 49‐year‐old male patient. Whole‐exome sequencing (WES) and RNA sequencing (RNAseq) were performed on 15 and 6 metastases, respectively. The comprehensive genomic and transcriptomic data generated in this report offer a unique opportunity to study the mechanistic origin and evolution of CUPs.

WES analysis identified a total of 748 nonsynonymous mutations in the 15 metastases, 276 of them were fully shared. Interestingly, the genetic similarity among all metastases pairs ranged from 58 to 82%, revealing a low degree of inter‐metastases heterogeneity. A similar scenario has recently been described in treatment‐naive metastases from patients with tumors of known primary, where 66% of the mutations per patient were clonal (homogeneous) among metastases (Reiter *et al*, 2019). Genetic homogeneity seems to be the rule rather than the exception in both scenarios, leaving room for the identification of potentially druggable targets to simultaneously eradicate multiple lesions. Moreover, considering the aggressive nature and rapid clinical course of CUPs, the presence of strong clonal oncogenic driver mutations could also explain the lack of heterogeneity in the CUP metastases.

Genomic data of several metastases from the same CUP patient are also an optimal setting to infer tumor evolutionary dynamics. Since the complexity of cancer arises from evolutionary processes of mutations, genetic drift, and selection, use of genomic sequencing is an invaluable tool to understand temporal and spatial patterns of somatic evolution (Turajlic *et al*, 2019). Although in Benvenuti *et al* (2020) methodological limitations related to sample type (bulk sequencing) and depth of coverage (< 50× in about 60% of targeted base pairs) could diminish the accuracy of their inferences, the authors suggest that the phylogenetic tree reconstruction of CUP metastases is consistent with the presence of a common cell of origin with stem‐like features that disseminate its progeny, an attractive hypothesis to explain CUP origin that merits further investigation.

The existence of cancer stem cells, first described in hematopoietic tumors and later in several solid tumors, has also been proposed as a driver of metastasis. In the case of CUPs, stem cell attributes such as migratory ability, plasticity, and high renewal capacity could explain metastatic dissemination without the requirement of establishing a primary tumor. CUP metastases might originate from stem cells or non‐stem cells that have acquired these biological properties due to DNA alterations, and are able to settle and colonize different organs. The phenotypic plasticity and undifferentiated state of these cells could also be behind the unpredictable metastatic patterns observed in CUPs, in contrast to the metastasis organotropism of cancers of known primary.

An additional layer of information, the transcriptomes of six CUP metastases, has also been analyzed by Benvenuti *et al* (2020). To our knowledge, this is the most comprehensive series of metastases from a case of CUP reported in the literature and is *per se* a valuable contribution to scientific knowledge.

Transcriptomic analysis of the CUP metastases revealed a lack of correlation with The Cancer Genome Atlas (TCGA) datasets of 33 different tumor types, a finding certainly unexpected. Although the unique features of CUPs could arise from a distinctive transcriptome, this conclusion contrasts with the achievement of CUP classifiers based on tissue‐specific expression profiles (Moran *et al*, 2017). Further studies to support this finding are encouraged, as well as inclusion of series of stem cells/stem‐like cells in the correlation analysis. Besides, additional caution should be taken considering that CUP samples analyzed in this study derived from warm autopsy.

Research autopsy approach holds a tremendous potential to contribute to understand physiological and pathological conditions. However, even over relatively short timeframes, death introduces a bias in cellular transcriptomes. Using mRNA sequencing data from multiple tissues of post‐mortem donors obtained from the Genotype Tissue Expression (GTEx) database, a recent systematic study found that the post‐mortem interval (PMI) impacts RNA stability in a very tissue‐dependent manner (Ferreira *et al*, 2018). The comparison of transcriptomes of TCGA normal tissue counterparts with normal tissues obtained at warm autopsy of the same CUP patient could further support the unique transcriptomic profile of the CUP metastases reported by the authors.

Multicentric, multidisciplinary, and multiomics studies are needed to decipher the biological and clinical complexity of CUPs. With this aim, the TCGA program, the major effort to catalog the molecular alterations in cancer, has launched a multiomic project under our coordination to characterize CUPs encompassing genomic, transcriptomic, and epigenomic data. This project aims at expanding our knowledge on the molecular mechanisms governing CUP carcinogenesis and will also be key to assess the existence of different CUP entities, and to identify potential therapeutic targets that can be translated to clinical practice. Finally, increasing examples of successful use of immunotherapies in several tumor types encourages the scientific community to explore this approach to improve the dismal outcome of CUP patients.

## Conflict of interest

ME is a consultant of Ferrer International and Quimatryx.
